# Establishing reference values for the match running performances of thirteen specific positional roles at UEFA Euro 2024

**DOI:** 10.5114/biolsport.2025.148535

**Published:** 2025-03-24

**Authors:** Shuyao Chen, Piotr Zmijewski, Paul S Bradley

**Affiliations:** 1 Typewind Ltd, UK; 2 Jozef Pilsudski University of Physical Education in Warsaw, 00-809 Warsaw, Poland; 3 Research & Development Centre Legia Lab, Legia Warszawa, Poland; 4 Football Science Consultant, UK

**Keywords:** Soccer, Match analysis, Position, High-intensity, Peak period, Speed, International

## Abstract

This study aimed to (1) establish reference values for the match running performances of thirteen specialised positions at the men’s UEFA Euro’s 2024, (2) examine positional performances across various game periods and (3) add various layers of match context to the demands by combining physical, technical, tactical and individual information. All fifty-one games at the tournament were evaluated by analysing optical tracking data alongside match events. During matches, central and defensive midfielder subsets covered more total distance than other positions (P < 0.01; Effect Size [ES]: 1.4–3.1). Wide defender subsets in addition to attacking midfielders and forwards playing as a pair covered greater high-intensity running and sprinting distance (≥20.0 and ≥25.0 km · h^−1^) than centre back subsets, defensive midfielders and lone forwards (P < 0.01; ES: 0.9–2.7 and 1.3–2.1). The peak high-intensity running distance in a 1-min period was greater for wide defender subsets compared to centre backs playing in a four-defender system (P < 0.01; ES: 0.9–1.2). More solo runs into key play areas occurred for attacking midfielders and wide forwards than centre back subsets (P < 0.01; ES: 0.9–2.1). Wide forwards performed more solo runs into the penalty area than other positions (P < 0.05; ES: 0.9–2.0). While centre back subsets recovered the ball more often compared to wide defenders, central and attacking midfielders in addition to forward subsets (P < 0.01; ES: 0.8–2.2). These findings provide contemporary evidence regarding the specific positional demands encountered during international matches. This information could serve as a basic framework for practitioners when developing position-specific training.

## INTRODUCTION

The match demands of football are multifaceted whereby players perform unpredictable movements alongside an array of technical and tactical actions [1]. Match analysis can be a useful tool to quantify such activities in an attempt to evaluate performances [2]. This can subsequently be used by practitioners to provide insights and aid in the development of football-specific drills [3]. Moreover, analysing various competitions can help to establish relevant benchmarks across selected metrics [4–6]. Researchers have previously examined the match demands of major international tournaments such as the FIFA World Cup 2014, 2018 and 2022 editions [7–9]. Although the men’s UEFA Euro’s have yet to be benchmarked from a match demands perspective. To accommodate for this, a study that examines the most recent edition of this tournament could establish up-to-date reference values across numerous match indices. A detailed examination of this tournament would also provide a baseline from which performance changes could be monitored in future. This is especially important given the intensification of modern match demands in both domestic and international competitions [4, 5, 10].

A consistent finding in football science across the last four to five decades relates to the unique positional demands placed on players within the team [3, 11–14]. This work reveals some well-established trends but also indicates that selected roles are evolving due to modern tactical developments [4, 5]. For instance, central midfielders typically cover the most overall ground during matches, as these roles are highly active during all phases of play [5, 7]. However, positional trends for high-intensity metrics are much more variable within the literature. Traditionally, wide midfielders usually cover the greatest distance at the higher intensities [5, 13, 15]. However, contemporary trends have identified the evolving high-intensity nature of wide defender subsets such as wing backs [16]. Additionally, centre backs have consistently been found to be the least demanding role in the team [5, 7]. Although, recent work has illustrated subtle physical and tactical differences between centre backs playing in a three versus a four at the back [17]. Therefore, an in-depth examination of up to thirteen specialised positions during a recent international tournament could provide valuable insights into evolving roles of modern players. Moreover, a study that also breaks positional performance down into peak periods of the game (e.g., 1-min periods) could aid the development of position-specific training. Despite the high variability inherent in peak match-play periods [18], practitioners can still benefit from viewing these periods qualitatively (e.g., video) and quantitatively (e.g., tracking data). This can allow context to be applied into position-specific training tasks that create an appropriate physical, technical and tactical stimulus [19]. The 1-min peak periods of match-play also align well with the work durations used in speed endurance training [20, 21].

Football performances are highly linked to the tactical and technical components of match-play [22]. Thus, combining match event data alongside physical analyses may provide much needed context and aid interpretation [23]. Previous work provided a basic breakdown of technical and tactical indices across five positions [5]. Although a more comprehensive array of modern metrics are currently available to investigate technical and tactical patterns at major tournaments [24, 25]. Moreover, limited information exists on detailed position-specific match performance trends using metrics such as solo runs into crucial pitch areas in addition to ball recoveries. Such in-depth analyses may provide beneficial information to practitioners regarding the types of technical and tactical actions and/or scenarios to include in position-specific drills [14]. Contextual information can also be derived by highlighting pertinent individual examples in selected positions and matches, particularly at the upper and lower extremes of various performance metrics. Using a combination of contextual information from technical and tactical metrics alongside relevant examples may aid our understanding of the dynamic interplay between performance factors.

Therefore, this study aimed to (1) establish reference values for the match running performances of thirteen specialised positions at the men’s UEFA Euro’s 2024, (2) examine positional performances in various game periods and (3) add various layers of match context to the demands by combining physical, technical, tactical and individual information.

## MATERIALS AND METHODS

### Sample and Match Analysis System

Fifty-one matches from twenty-four teams at the men’s UEFA Euro’s 2024 tournament were evaluated by analysing data from an optical tracking system (Hawk-Eye Innovations Ltd, Basingstoke, UK). This system’s validity has been independently verified against gold standard methods [26]. Positional roles were manually assigned to players using video footage alongside tactical analyses (e.g., average pitch position, team formation and style of play). To standardise to previous work [7], players were only included if they completed the entire match (e.g., 90-min plus added time but not extra time). This allowed 544 player observations to be analysed across thirteen specialised positions (3 ± 1 matches per player). Defenders were subdivided into five roles: (122 centre backs in a four-defender formation [CB^4^], 55 outside centre backs of a three-defender formation [CB^3O^], 31 centre backs in the middle of a three-defender formation [CB^3C^], 92 full backs in a four-defender formation [FB] and 31 wing backs in a three-defender formation [WB]). While midfielders were segmented into five roles: (59 defensive midfielders [DM], 12 single pivot midfielders [DM^1^], 38 central midfielders [CM], 6 wide midfielders [WM], 45 attacking midfielders [AM]). Finally, forwards were partitioned into three roles: (18 wide forwards [WF], 21 lone forwards [FW^1^] and 14 forwards playing as a pair [FW^2^]). Most of the secondary data presented were freely available [27] and arose as a condition of the players involvement in the tournament [28], thus no ethical clearance was required. Data across selected parameters (e.g., high-intensity distance) were not available via the source above and could be shared by the authors upon a reasonable and well justified request (final decision will be at the discretion of the authors).

### Physical Metrics

Players’ activities were coded into the following categories and speed thresholds: standing (0.0–0.9 km · h^−1^), walking (1.0–6.9 km · h^−1^), jogging (7.0–14.9 km·h^−1^), running (15.0–19.9 km·h^−1^), high-speed running (20.0–24.9 km · h^−1^) and sprinting (≥25.0 km · h^−1^). Data were then separated into three main physical metrics: total, high-intensity running and sprinting distance (e.g., presented as absolute values). Total distance represented the sum of the ground covered in all speed thresholds and categories above. High-intensity running consisted of the aggregated distance covered in the high-speed and sprinting categories (≥20.0 km · h^−1^), while sprinting included the distance covered at the highest speed threshold (≥25.0 km · h^−1^). The distance covered in each of these metrics above were obtained at 45-min time periods plus added time to examine half-by-half changes (e.g., presented as relative values). Moreover, the peak 1-min period of high-intensity running during match-play for each position was examined using a rolling average [29]. Top speeds attained in games were also quantified across all positions. Regarding the above metrics, it is important for the reader to be aware that the raw data was individually clipped and re-filtered based on the present studies inclusion criteria and filtering specifications. Thus, this process may cause some differences compared to UEFA trends [27].

### Technical and Tactical Metrics

To further contextualise the physical data, the present method also evaluated the technical and tactical metrics during match-play. This included the coding of distribution, touch, offensive and defensive match events (Table 1).

**TABLE 1:** Match events for each specialised positional role at UEFA Euro 2024.

Variables	CB^4^	CB^O3^	CB^C3^	FB	WB	DM	DM^1^	CM	WM	AM	WF	FW^1^	FW^2^	Basic Summary of P-values
*Distribution Events*														

Total Passes (No)	61.7 ± 24.3	51.2 ± 21	52.3 ± 21.5	48.3 ± 19.4	36.5 ± 12.2	63.4 ± 23.8	61.4 ± 31.1	49.6 ± 19.8	43.5 ± 13.0	41.0 ± 16.4	36.4 ± 16.2	19.9 ± 9.0	16.4 ± 7.5	FW^1*^, FW^2^<All^#^Except WB, WM, WF. DM, DM^1^>FW^1*^, FW^2*^.WB^*^, WF^*^.
Passes Completed (%)	91.3 ± 7.0	87.6 ± 7.4	91.3 ± 6.4	86.8 ± 8.2	78.5 ± 10.1	91.4 ± 6.7	88.0 ± 7.6	87.2 ± 7.1	88.6 ± 8.5	83.4 ± 8.9	78.2 ± 13.5	80.9 ± 12.2	76.1 ± 13.1	DM, CB^4^, CB^3c^>AM^*^, FW^1*^, FW^2*^. WB^*^, WF^*^. CB^3o^, CM>FW^2*^. WB^*^, WF^*^.
Total Pass Forward (No)	19.9 ± 9.8	19.9 ± 9.8	16.7 ± 7.0	15.6 ± 6.9	13.0 ± 6.1	17.3 ± 9.2	15.5 ± 6.2	14.0 ± 6.5	11.0 ± 2.1	10.4 ± 5.4	7.9 ± 4.4	4.1 ± 3.2	3.0 ± 2.5	FW^1*^, FW^2^<All^#^Except WM, WF. CB^4^, CB^3o^, CB^3c^>AM^*^,CM^#^, WB^#^, WF^#^.
Pass Forward Completed (%)	79.5 ± 13.5	77.1 ± 15.0	81.3 ± 12.7	75.6 ± 16.5	64.7 ± 18.7	80.7 ± 16.2	77.16 ± 13.8	73.0 ± 15.5	77.2 ± 15.4	68.2 ± 21.2	62.71 ± 22.9	62.2 ± 31.5	56.9 ± 39.2	FW^1*^, FW^2^<CB^4*^, CB^3o#^, CB^3c^^*^, DM^*^.
Total Crosses (No)	0.2 ± 0.6	0.7 ± 0.9	0.1 ± 0.3	2.2 ± 2.2	2.7 ± 2.9	1.1 ± 2.3	0.5 ± 0.9	1.4 ± 1.5	4.2 ± 3.9	3.6 ± 3.6	3.8 ± 2.4	1.0 ± 1.6	1.0 ± 1.2	WF, AM, WM>CB^4*^, CB^3o^^#^, CB^3c^^*^, DM^*^, DM^1*^, CM, ^*^ FW^1*^, FW^2*^

Ball Touch Events														

Dribbling (No)	0.3 ± 0.6	0.4 ± 0.8	0.1 ± 0.3	1.0 ± 1.4	1.5 ± 1.6	0.9 ± 1.1	0.7 ± 0.5	0.9 ± 1.2	2.0 ± 1.4	2.4 ± 1.9	4.3 ± 3.7	1.9 ± 1.7	2.9 ± 2.6	WF, FW^2^, AM>CB^4*^, CB^3o^^*^, CB^3c^^*^, DM^*^, DM^1*^, CM^*^, FB^*^.
Total Ball Touches (No)	121.3 ± 44.4	105.4 ± 37.4	102.1 ± 40.0	106.5 ± 36.7	85.5 ± 23.0	122.0 ± 46.2	115.7 ± 52.9	98.4 ± 38.0	93.8 ± 23.0	89.5 ± 28.8	83.3 ± 31.8	49.7 ± 19.1	44.6 ± 17.4	CB^4^, DM>FW^1*^, FW^2*^, WB^*^, WF^*^. CB3O^*^, CB3C^*^, DM^*^, DM^1*^, FB^*^>FW^1*^, FW^2*^.
One Touch Play (No)	11.9 ± 4.8	11.4 ± 5.3	10.8 ± 4.3	10.9 ± 4.6	10.4 ± 4.4	14.8 ± 5.4	17.6 ± 7.1	14.3 ± 5.1	9.2 ± 5.5	11.3 ± 5.9	7.9 ± 5.1	7.1 ± 4.1	7.1 ± 2.9	DM, DM^1^>AM^#^, CB^4^^#^, CB^3o^^#^, CB^3c^^#^, FB^*^, FW^1*^, FW^2*^, WB^*^, WF^*^. CM>FB^#^, FW^1*^, FW^2*^

*Offensive Events*														

Total Shots (No)	0.5 ± 0.7	0.5 ± 0.8	0.5 ± 0.8	0.4 ± 0.7	0.4 ± 0.7	1.1 ± 1.1	0.5 ± 0.7	1.3 ± 1.2	1.2 ± 1.2	2.3 ± 1.7	2.5±1.9	3.3 ± 2.1	2.3 ± 1.5	WF, AM, WM, FW^1*^, FW^2^>CB^4*^, CB^3o^^*^, CB^3c^,^*^ DM^*^, DM^1*^, CM^*^, FB^*^.
Shots on Target (No)	0.1 ± 0.4	0.1 ± 0.4	0.1 ± 0.4	0.1 ± 0.3	0.1 ± 0.4	0.3 ± 0.5	0.1 ± 0.3	0.5 ± 0.9	0.3 ± 0.5	0.6 ± 0.8	0.9 ± 0.9	1.4 ± 1.4	0.6 ± 0.5	FW^1^, WF>CB^4*^, CB^3o^^*^, CB^3c^^*^, DM^*^, DM^1*^,FB^*^, WB. FW^1^>FW^2^^#^.
Solo Runs: Attacking Third (No)	0.8 ± 1.2	1.1 ± 1.5	0.3 ± 0.5	1.6 ± 1.5	1.4 ± 1.2	1.4 ± 1.5	1.3 ± 1.4	1.8 ± 1.9	1.2 ± 1.2	2.1 ± 1.9	2.2 ± 1.8	1.1 ± 0.8	1.8 ± 2.3	CB^4^, CB^3c^<AM^*^, CM^#^, FB^*^, WF^#^. CB^3o^<AM^#^.
Solo Runs: Key Play Area (No)	0.6 ± 1.3	0.6 ± 1.0	0.1 ± 0.5	0.9 ± 1.2	0.6 ± 0.8	1.4 ± 1.7	1.3 ± 1.6	1.5 ± 1.8	1.3 ± 1.5	2.0 ± 1.9	2.6 ± 1.6	1.2 ± 1.7	1.4 ± 2.2	CB^4^, CB^3c^, CB^3o^<AM^*^, WF^*^.
Solo Runs: Penalty Area (No)	0.0 ± 0.1	0.1 ± 0.2	0.0 ± 0.0	0.3 ± 0.6	0.3 ± 0.7	0.2 ± 0.5	0.0 ± 0.0	0.4 ± 0.6	1.0 ± 0.9	0.9 ± 1.0	1.9 ± 1.3	0.7 ± 1.4	0.7 ± 0.8	WF>All^*^ Except WM.

*Defensive Events*														

Ball recovery (No)	5.6 ± 2.7	5.1 ± 2.3	6.4 ± 2.7	4.9 ± 2.3	3.7 ± 2.4	4.8 ± 2.4	4.6 ± 2.9	3.2 ± 2.0	2.8 ± 1.7	2.2 ± 1.5	1.4 ± 1.2	0.8 ± 0.9	1.0 ± 0.9	CB^4^, CB^3o^, CB^3c^>WB^*^, CM^*^, AM^*^, WF^*^, FW^1*^, FW^2*^.
Tackles (No)	1.1 ± 1.1	1.4 ± 1.1	1.0 ± 0.9	1.3 ± 1.4	1.5 ± 1.4	1.8 ± 1.4	1.8 ± 1.5	1.8 ± 1.5	1.7 ± 0.8	1.2 ± 1.3	0.8 ± 1.0	0.1 ± 0.4	0.7 ± 1.2	FW^1^<CB^3o^^*^, FB^#^, WB^#^, DM^*^, DM^1^^#^, CM^*^^#^.
Aerial duel (No)	1.5 ± 1.4	1.8 ± 1.5	2.0 ± 1.8	0.8 ± 1.0	1.0 ± 1.1	1.0 ± 1.2	0.5 ± 0.5	0.6 ± 1.1	0.2 ± 0.4	0.8 ± 1.3	0.6 ± 0.7	1.7 ± 2.0	1.4 ± 1.3	CB^4^, CB^3o^, CB^3c^>CM^*^, DM^*^, AM^*^, WF^#^

All values have been rounded up or down to one decimal place. *P*<0.05^#^; *P*<0.01^*^

### Statistical Analyses

Data were filtered and visualised using Python (Version 3.11, Python Software Foundation, USA), while SPSS was used for statistical analyses (Version 26.0, SPSS Inc, IBM Corp, USA). Descriptive statistics were calculated on each variable. To verify normality, z-scores were obtained through dividing the skewness and kurtosis values by their standard error. Positional differences across metrics for full-match and peak 1-min periods were determined using one-way analysis of variance (ANOVA). In the event of a significant difference occurring, univariate analyses using Bonferroni-corrected pairwise comparisons were employed. Half-by-half positional performances were evaluated using Bonferroni-corrected paired sample *t*-tests. Statistical significance was set at *P* < 0.05. Quadrant plot analyses composed of a simple percentage distribution computation. The intra-positional coefficient of variation (CV) was used to determine the data spread across metrics. Cohen’s *d* effect sizes (ES) were computed to determine the meaningfulness of any differences. The ES magnitudes were classed as trivial (< 0.2), small (> 0.2–0.6), moderate (> 0.6–1.2) and large (> 1.2). Pearson’s coefficients were used for correlation analyses and their magnitudes were regarded as trivial (*r* < 0.1), small (*r* > 0.1–0.3), moderate (*r* > 0.3–0.5), large (*r* > 0.5–0.7), very large (*r* > 0.7–0.9), and nearly perfect (*r* > 0.9). Values are presented as means and standard deviations unless otherwise stated.

## RESULTS

### Reference Values and Variation

During matches, CM in addition to DM^1^ and DM covered more total distance than other positions (Figure 1A; *P* < 0.01; ES: 1.4–3.1), except for WB, WM and AM. Figure 1A also demonstrates that WF, FW^1^ and FW^2^ displayed more variation for total distance covered (CV: 8.0–9.4%) than CB^3C^, CB^3O^, CB^4^, DM and CM (CV: 5.2–6.0%). Moreover, FB, WB, AM and FW^2^ covered greater high-intensity running and sprinting distance (≥20.0 and ≥25.0 km · h^−1^) than CB^3C,^ CB^3O^, CB^4^, DM and FW^1^ (Figures 1B and 1C; *P* < 0.01; ES: 0.9–2.7 and 1.3–2.1). Figures 1B and 1C also revealed that CB^3C^, CB^3O^, CB^4^ and DM^1^ displayed more variation (CV: 30.3–36.1% and 49.0–69.1%) for high-intensity running and sprinting distance compared to WB, WM, WF and FW^1^ (CV: 19.6–24.6% and 29.9–43.2%). Top speeds attained in games were greater for FB, WB, FW^1^ and FW^2^ than CB^3C^, CB^3O^ and DM (Figure 1D; *P* < 0.05; ES: 0.7–1.6). Moreover, Figure 1D revealed that FB and WB made up six of the top ten sprint speeds attained at the tournament (34.8–35.7 km·h^−1^), while three were from FW^1^ or FW^2^ (34.8–35.1 km · h^−1^).

**FIG. 1A:**
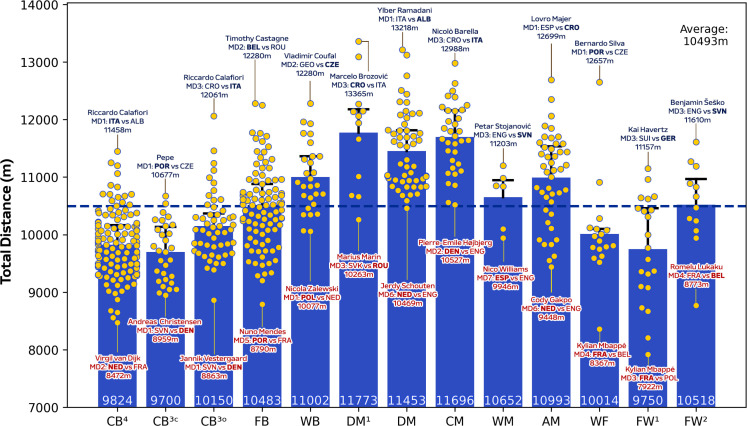
Total distance and variation for each positional role at Euro 2024. Data are normalized for 90 min plus added time (excludes goalkeepers and extra time) and are presented as absolute values. Centre backs in a four-defender formation (CB^4^), outside centre backs of a three-defender formation (CB^3O^), centre backs in the middle of a three-defender formation (CB^3C^), full backs in a four-defender formation (FB), wing backs in a three-defender formation (WB), defensive midfielders (DM), single pivot midfielders (DM^1^), central midfielders (CM), wide midfielders (WM), attacking midfielders (AM), wide forwards (WF), lone forwards (FW^1^) and forwards playing as a pair (FW^2^). Black = highest value per position, red = lowest value per position. Examples highlight both the individual player and the specific match. The dotted line denotes the mean for all tactical roles.

**FIG. 1B:**
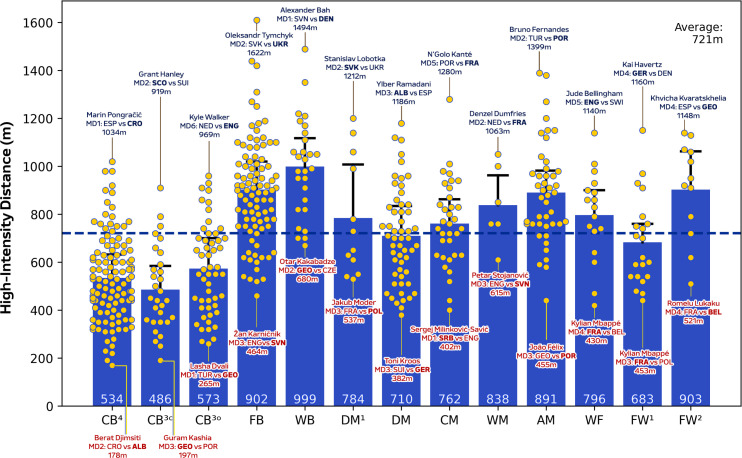
High-intensity distance (≥20 km⋅h^-1^) and variation for each positional role at Euro 2024. Data are normalized for 90 min plus added time (excludes goalkeepers and extra time) and are presented as absolute values. Centre backs in a four-defender formation (CB^4^), outside centre backs of a three-defender formation (CB^3O^), centre backs in the middle of a three-defender formation (CB^3C^), full backs in a four-defender formation (FB), wing backs in a three-defender formation (WB), defensive midfielders (DM), single pivot midfielders (DM^1^), central midfielders (CM), wide midfielders (WM), attacking midfielders (AM), wide forwards (WF), lone forwards (FW^1^) and forwards playing as a pair (FW^2^). Black = highest value per position, red = lowest value per position. Examples highlight both the individual player and the specific match. The dotted line denotes the mean for all tactical roles.

**FIG. 1C:**
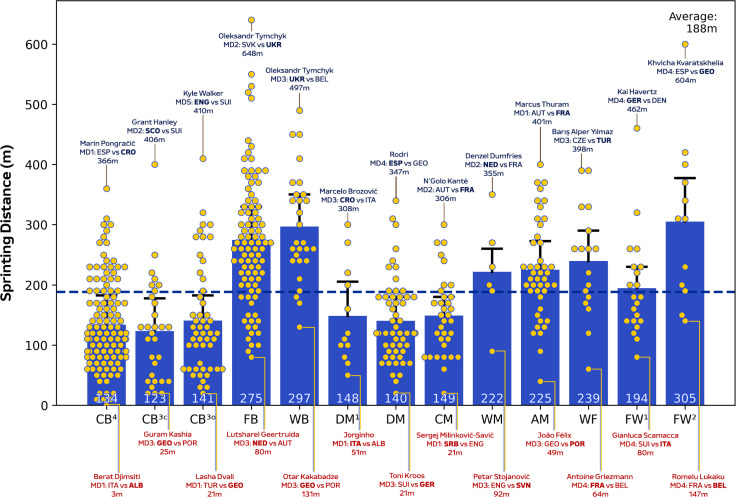
Sprint Distance (≥25 km⋅h^-1^) and variation for each positional role at Euro 2024. Data are normalized for 90 min plus added time (excludes goalkeepers and extra time) and are presented as absolute values. Centre backs in a four-defender formation (CB^4^), outside centre backs of a three-defender formation (CB^3O^), centre backs in the middle of a three-defender formation (CB^3C^), full backs in a four-defender formation (FB), wing backs in a three-defender formation (WB), defensive midfielders (DM), single pivot midfielders (DM^1^), central midfielders (CM), wide midfielders (WM), attacking midfielders (AM), wide forwards (WF), lone forwards (FW^1^) and forwards playing as a pair (FW^2^). Black = highest value per position, red = lowest value per position. Examples highlight both the individual player and the specific match. The dotted line denotes the mean for all tactical roles.

**FIG. 1D:**
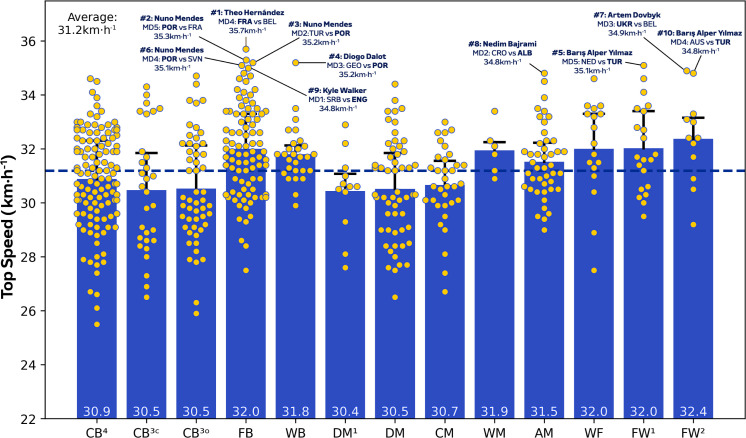
Top speed and variation (including top ten speeds) for each positional role at Euro 2024. Data are normalized for 90 min plus added time (excludes goalkeepers and extra time) and are presented as absolute values. Centre backs in a four-defender formation (CB^4^), outside centre backs of a three-defender formation (CB^3O^), centre backs in the middle of a three-defender formation (CB^3C^), full backs in a four-defender formation (FB), wing backs in a three-defender formation (WB), defensive midfielders (DM), single pivot midfielders (DM^1^), central midfielders (CM), wide midfielders (WM), attacking midfielders (AM), wide forwards (WF), lone forwards (FW^1^) and forwards playing as a pair (FW^2^). Numbers = top ten sprint speeds in the tournament. Examples highlight both the individual player and the specific match. The dotted line denotes the mean for all tactical roles.

### Quadrant Plots

Figures 2A-C correlate two dimensions of physical performance using quadrant plots to compare specialist positions. Regarding defenders, Figure 2A indicates all CB subsets demonstrated correlations between total and high-intensity running distance (≥20.0 km · h^−1^). The associations were moderate for CB^3O^ and CB^3C^ (*r* = 0.32 and 0.42; *P* < 0.05) and large for CB^4^ (*r* = 0.51; *P* < 0.01). Both CB^3C^ and CB^4^ occupied the lower-left quadrant more versus CB^3O^ (LLQ: 86.7% and 77.5% vs 67.3%). While CB^3O^ occupied the upper-right (URQ: 7.7% vs 3.3% and 5.0%) and lower-right quadrants more (LRQ: 13.5% vs 3.3% and 7.5%) versus CB^3C^ and CB^4^. Large associations were also observed between total and high-intensity running distance for both a FB and WB (*r* = 0.54 and 0.52; *P* < 0.01). Although WB residing more in the upper-right quadrant than FB (URQ: 71.4% vs 47.3%). Regarding midfielders, Figure 2B indicates associations were small for CM and WM (*r* = 0.15 and -0.11; *P* > 0.05), moderate for DM (*r* = 0.39; *P* < 0.01) and DM^1^ (*r* = 0.42; *P* > 0.05), in addition to large for AM (*r* = 0.54; *P* < 0.01) between total and high-intensity running distance. Interestingly, DM, DM^1^ and CM resided more on the right side of the plot compared to others (URQ: 45.5%, 50.0% and 55.9%; LRQ: 52.7%, 41.7% and 44.1%). Trends for AM exhibited the highest proportion in the upper-right quadrant (URQ: 68.2%), while WM primarily occupied the upper-right and left quadrants (URQ: 50.0% and ULQ: 33.3%). Regarding forwards, Figure 2C found moderate associations for WF and FW^2^ (*r* = 0.38 and 0.35; *P* > 0.05), while it was large for FW^1^ (*r* = 0.66; *P* < 0.01) between total and high-intensity running distance. Moreover, WF were primarily distributed in the upper-left quadrant (ULQ: 64.7%), while FW^1^ were mainly found in the lower-left quadrant (LLQ: 57.1%) and FW^2^ were largely found in the upper-right quadrant (URQ: 50.0%).

**FIG. 2A:**
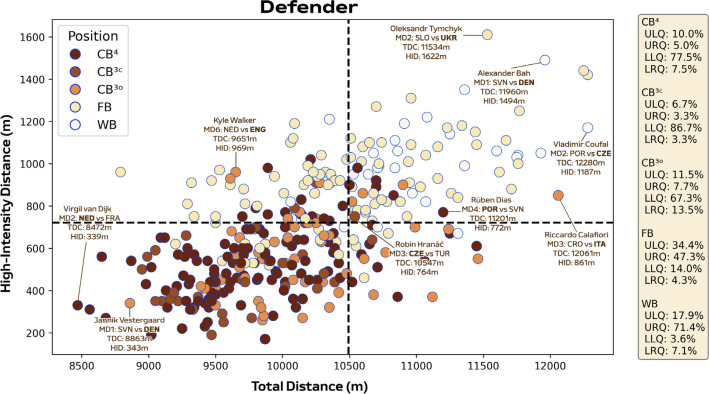
Defenders total distance (TDC) versus high-intensity distance (≥20 km·h^-1^; HID) at Euro 2024. Data are normalized for 90 min plus added time (excludes goalkeepers and extra time) and are presented as absolute values. Crosshairs were based on the mean for all tactical roles. Centre backs in a four-defender formation (CB^4^), outside centre backs of a three-defender formation (CB^3O^), centre backs in the middle of a three-defender formation (CB^3C^), full backs in a four-defender formation (FB), wing backs in a three-defender formation (WB). Data on the right highlights the percentages of player observations in the various quadrants: ULQ = upper-left quadrant, URQ = upper-right quadrant, LLQ = lower-left quadrant, LRQ = lower-right quadrant. Examples highlight both the individual player and the specific match.

**FIG. 2B:**
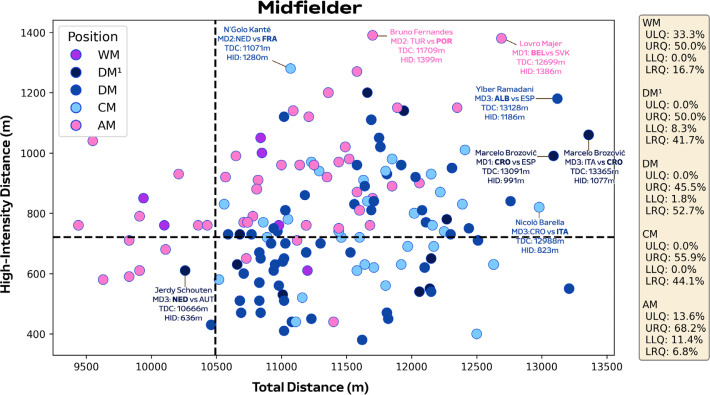
Midfielders total distance (TDC) versus high-intensity distance (≥20 km·h^-1^; HID) in Euro 2024. Data are normalized for 90 min plus added time (excludes goalkeepers and extra time) and are presented as absolute values. Crosshairs were based on the mean for all tactical roles. Defensive midfielders (DM), single pivot midfielders (DM^1^), central midfielders (CM), wide midfielders (WM), attacking midfielders (AM). Data on the right highlights the percentages of player observations in the various quadrants: ULQ = upper-left quadrant, URQ = upper-right quadrant, LLQ = lower-left quadrant, LRQ = lower-right quadrant. Examples highlight both the individual player and the specific match.

**FIG. 2C:**
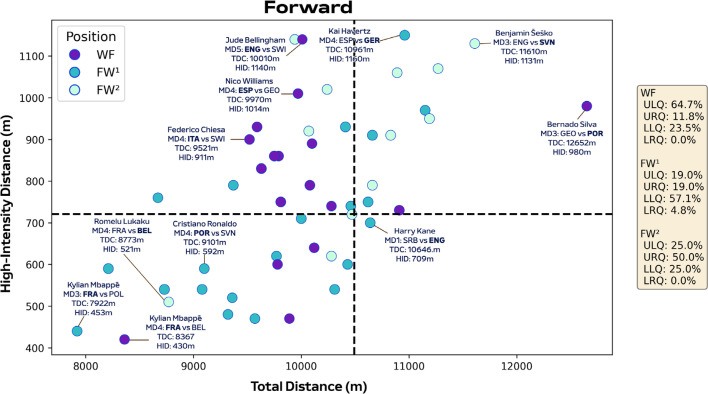
Forwards total distance (TDC) versus high-intensity distance (≥20 km·h^-1^; HID) at Euro 2024. Data are normalized for 90 min plus added time (excludes goalkeepers and extra time) and are presented as absolute values. Crosshairs were based on the mean for all tactical roles. Wide forwards (WF), lone forwards (FW^1^) and forwards playing as a pair (FW^2^). Data on the right highlights the percentages of player observations in the various quadrants: ULQ = upper-left quadrant, URQ = upper-right quadrant, LLQ = lower-left quadrant, LRQ = lower-right quadrant. Examples highlight both the individual player and the specific match.

### Match-Play Periods

Most positions demonstrated a second half reduction on a per minute basis for total distance covered compared to the first half (*P* < 0.05; ES: 0.5–1.3), with the only exception being DM^1^, WB and WM. Small to moderate declines occurred between halves for high-intensity running distance on a per minute basis (≥20.0 km · h^−1^) across positions (*P* > 0.05; ES: 0.1–0.4), with the only exception being DM and WB. Most positions maintained their sprinting distance (≥25.0 km · h^−1^) across halves on a per minute basis (*P* > 0.05; ES:0.1–0.4). Albeit non-significant, FB and WB actually increased their second half sprinting outputs versus the first half. Peak 1-min high-intensity running distance for FB and WB was greater versus CB^4^ (69.0 ± 24.0 and 74.4 ± 22.0 vs 50.8 ± 17.4 m) during the tournament (*P* < 0.01; ES: 0.9–1.2). Other positions covered between 52.8–63.2 m during this peak 1-min period (CB^3C^: 54.1±16.1, CB^3O^: 58.1 ± 22.4, DM: 61.3 ± 22.7, DM^1^: 52.8 ± 20.5, CM: 62.4±15.1, AM: 63.2±14.7, WM: 61.0±16.7, WF: 57.0±13.4, FW^1^: 54.8 ± 13.0 and FW^2^: 58.5 ± 22.9 m).

### Technical and Tactical Metrics

Table 1 presents position-specific match events at UEFA Euro 2024. Regarding distribution events, DM and DM^1^ passed more than WB, WF, FW^1^ and FW^2^ (*P* < 0.01; ES: 1.1–2.7). Total crosses were greater for WF, AM, WM than CB^4^, CB^3O^, CB^3C^, DM, DM^1^, CM, FW^1^, FW^2^ (*P* < 0.05; ES: 1.3–2.2). Selected ball touch events such as dribbling were higher for WF, FW^2^, AM than CB^4^, CB^3O^, CB^3O^, DM, DM^1^, CM, and FB (*P* < 0.01; ES: 0.7–1.7). Regarding offensive events, WF, AM, WM, FW^1^, FW^2^ performed more shots than CB^4^, CB^3O^, CB^3C^, DM, DM^1^, CM and FB (*P* < 0.01; ES: 0.8–1.9). Interestingly, FW^1^ had more shots on target than FW^2^ (*P* < 0.05; ES: 0.8). Moreover, AM, CM, FB and WF performed more solo runs in the attacking third compared to CB^4^ and CB^3C^ (*P* < 0.05; ES: 0.6–1.5). Solo runs into key play areas were greater for AM and WF than all three CB roles (*P* < 0.01; ES: 0.9–2.1). Interestingly, WF produced more solo runs into the penalty area than other positions except WM (*P* < 0.05; ES: 0.9–2.0). In respect to defensive events, CB^4,^ CB^3O^ and CB^3O^ recovered the ball more often than WB, CM, AM, WF, FW^1^ and FW^2^ (*P* < 0.01; ES: 0.8–2.2).

## DISCUSSION

The current study was the first to benchmark the match demands at the men’s UEFA Euro 2024 tournament across specialised positions and selected game periods. This investigation also breaks new ground by harnessing a context-centric approach which adds a much-needed narrative to the data trends.

Strikingly similar averages were attained for players total distance covered at the UEFA Euro 2024 tournament compared to those at the FIFA World Cup 2022 [7]. Despite the upper speed thresholds being identical at both competitions (e.g., ≥20.0 and ≥25.0 km · h^−1^), the distances covered were lower in the present study. This disparity could be due to differences in the optical tracking systems employed, the filtering and dwell times applied to the raw data, in addition to varying match durations across both tournaments [30, 31]. Thus, tournament benchmark comparisons are challenging and this emphasises the necessity to develop correction equations that align data from various optical tracking systems. Despite this, position-specific similarities and differences between these two recent international tournaments were plentiful across metrics. For example, total distance covered was greatest for central and defensive midfielders (~11.4–11.9 km) and lowest for centre backs (~9.7–10.1 km) at both competitions. This is logical as midfielders are continually involved in most phases of play [7, 22]. While centre backs are more active during defensive phases as they are heavily impacted by the opposition’s offensive quality and work-rate [32]. Although variations exist due to the type of centre back role, and the style of play employed and even the calibre of the player. Outside centre backs differed hugely at the extremes for total distance covered. For example, Italy’s Riccardo Calafiori covering ~12.0 km against Croatia as he contributed to both defensive and attacking actions, with the latter including progressive carries into the opponent’s final third. This was in contrast to Denmark’s Jannik Vestergaard’s who covered ~8.8 km against Slovenia due to a more compact defensive display.

Position-specific trends at the higher intensities (≥20.0 and ≥25.0 km · h^−1^) were also comparable to that reported at the FIFA World Cup 2022 [7], with centre backs and defensive midfielders generally covering some of the lowest distances in both competitions (~500–800 m and 130–140 m, respectively). In contrast to the FIFA World Cup 2022, the present study revealed that full backs and particularly wing backs covered the most intense distance during games. Although it is crucial to mention that the FIFA data set did not subdivide wide defenders into full backs and wing backs [7]. Despite inconsistencies between international competitions, this latter finding concurred with those from the English Premier League that demonstrated the elevated match demands of wide defender subsets such as wing backs [16, 33]. Longitudinal data further support this finding as wide defenders were found to have evolved the most physically compared to other positions over a seven-season period [5]. Large correlation coefficients between total and high-intensity distances indicate that the modern wide defender may require volume and intensity characteristics in equal proportions. The high-intensity nature of a wing back was epitomised by the ~1500 m covered by Alexander Bah against Slovenia. This was related to his contributions across all phases of play, including simultaneous involvement in both attacking and defensive transitions within the same possession sequence. Interestingly, this is the first time this finding has been reported at the international level and thus adds further support towards the use of specialised positional labels when quantifying match demands.

The reader should be aware that significant differences across physical metrics were minimal between specialised subsets due to the substantial intra-positional variation for total (CV: ~5–10%), high-intensity running (CV: ~20–40%) and sprinting distances (CV: ~30–70%). Thus, the present investigation utilised quadrant plots to discern any nuanced differences between specialised roles. It was noticeable within this study that centre backs exhibited low volume and intensity characteristics (~67–87% in lower-left quadrant), while wide defenders displayed much more volume and intensity during matches (~47–71% in upper-right quadrant). Despite comparable trends in previous studies [7, 34], less is known regarding how specific central and wide defender subsets differ. Forcher et al. [17] found that central and wide defenders playing in a three versus a four at the back exhibited greater match demands (e.g., 3-5-2 vs 4-4-2 or 4-5-1). Similarly, the present data demonstrated that outside centre backs in a three-defender system were more likely to cover greater volume during matches than centre backs in a four-defender system (~14% vs 8% in lower-right quadrant). While wing backs in a three-defender system exhibited more intensity than full backs in a four-defender system (~71% vs 47% in upper-right quadrant). Playing three as opposed to four at the back may result in the outside centre backs being more volume-based as they are required to cover the ground of four defenders [17, 35]. The role of the outside centre back includes ball progression responsibilities during build-up phases, as evidenced by several noteworthy performances at UEFA Euro 2024 (e.g., Italy’s Riccardo Calafiori versus Croatia and Switzerland’s Ricardo Rodríguez against Germany). These performances exemplify the evolution of this role to contribute to offensive phases while also maintaining defensive responsibilities. Moreover, the defensive stability afforded by three versus four defenders and also the absence of a wide midfielder could free up wing backs to be more intense offensively compared to full backs [34]. Previous evidence suggests that wing backs cover more high-intensity distance than full backs while supporting teammates, overlapping, and attacking the space in behind [16]. In contrast, the present study found a similar number of events such as crosses and solo runs into offensive areas for both of the wide defender subsets. This disparity was likely to be related to the fact that solo runs and crosses are isolated events as opposed to integrated events that align physical-technical-tactical metrics. Despite wing backs being the most active position, it is important to note that it was actually a full back that was positioned the highest in the upper right corner of the quadrant plot (e.g., Ukraine’s Oleksander Tymchyk covered ~11.5 km in total and ~1600 m at high-intensity).

Central and defensive midfield subsets were found to be more volume-based as they resided more on the right side of the plot. Despite this, large individual variations occurred as Croatia’s Marcelo Brozović covered ~13.0 km in total and ~1000 m at high-intensity compared to ~10.7 km and ~600 m for the Netherlands Jerdy Schouten. This type of variation resulted in lower correlation coefficients between total and high-intensity game distances, but generally these midfield subsets were particularly volume dominant. The high work rate for these roles is understandable as they are usually very active during matches [7], as evidenced by a high event count for passes, ball touches, one-touch play, and tackles. While attacking midfielders were more intensity-based (~68% in upper-right quadrant) and offensive as shown by high event counts for crosses, dribbles, solo runs in attacking areas and shots on goal. These findings support previous work that have reported attacking midfielders performing more intense actions than defensive and central midfielders running with the ball, moving to receive, supporting teammates and also attacking the space in behind [16]. A plentiful number of these intense physical-tactical actions were linked to the high work rate of Portugal’s Bruno Fernandes against Turkey.

Regarding offensive positions, it was clear that lone forwards typically exhibited low volume and intensity characteristics compared to a forward pair that were more intensity-based (~57% vs 25% in lower-left and ~19% vs 50% in upper-right quadrants). While wide forwards displayed low volume but high-intensity characteristics compared to a single or a pair of forwards (~65% vs 19% or 25% in upper-left quadrant). These trends could be linked to the positional requirements of various tactical systems that employ wide forwards (e.g., 4-3-3/3-4-3), two forwards (e.g., 4-4-2/3-5-2) or a single forward (e.g., 4-5-1/4-2-3-1). Previous findings suggest that forwards playing in a front two or three are to a certain extent, taxed more physically than lone forward [36]. This could be due to forward pairs or trios feeding off each other’s intense movement and positionally interchanging regularly [34]. While lone forward are more static as they not only hold up play to bring teammates into the game but also have to be clinical in front of goal despite limited support. This latter point was corroborated by trends within the present study as shots on target were higher for lone forwards than those operating wide or as a pair. Although large variations existed for lone forwards in the present sample. For example, France’s game model enabled Kylian Mbappé freedom to exert himself accordingly, hence his low work rate. This differed from Germany’s more industrious Kai Havertz as his game was characterized by extensive defensive contributions and supportive movements in attacking phases. These contrasting approaches highlight the tactical diversity within the lone forward position at UEFA Euro 2024. The present findings demonstrate that players match demands are not only dependent upon their positional role but also on the formation and style of play employed by their team.

The present data also revealed that full backs and wing backs covered the greatest high-intensity distance during peak 1-min periods of matches (~70–75 m) and this was different from centre backs playing in a back four (~50 m). Comparable average and positional values during peak 1-min periods have been reported in domestic Danish, English and Spanish football [1, 37, 38]. No other differences were found signifying that most positions have to work intensely during selected moments of the game (~55–65 m). Thus, to enable players to repeatedly perform intense actions during match-play, it is crucial players perform high-intensity training regularly. For instance, speed endurance training has been found to be particularly beneficial at developing a player’s physical capacity [39]. Although this type of training can be successfully performed using generic running and football drills [20, 40], the present findings suggest the adoption of a position-specific approach. Although practitioners could use these trends to ensure selected positions achieve the equivalent output in selected drills, this approach lacks essential detail to develop drills [41]. Thus, it is imperative to draw from previous research regarding contextualised positional high-intensity efforts that occur in- and out-of-possession [16, 23, 37]. For example, in-possession forwards perform more intense efforts in the offensive third, whilst attacking the space in behind and breaking into the box [14]. Whilst wide defenders and midfielders produce regular intense efforts running down the channel, with wide defenders also completing a greater number of overlapping runs [16]. Out-of-possession, positions with a major defensive role in the team like central and wide defenders and defensive midfielders produce more intense efforts covering space and recovery running, whilst all forwards perform frequent efforts pressing the opposition. Thus, these contexts can be translated into position-specific conditioning drills using either collective patterns of play or individual isolated drills [21]. Although the present investigation included peak 1-min high-intensity running trends, the inclusion of peak mechanical data would have enhanced translation to drill design [42].

Prior to establishing conclusions, it is imperative that the reader is aware of some of the present studies shortcomings. Despite a large sample size overall, the sample per position was low for selected roles and this may have impacted trends (e.g., wide midfielders were particularly under-represented). Although ‘micro’ context was derived from individual examples, the current study lacked any direct integration of the physical-technical-tactical data. This limited the ‘macro’ contextual insights derived from the study and their implications for training. Thus, the alignment of data sources should be explored in future research. A multi-factorial approach that included accelerations/decelerations and directional changes would have provided more information regarding the specific demands of various positions [42]. Furthermore, the findings would have been enhanced if raw data were segmented based on effective playing time [43]. The present studies trends were singularly geared towards performance factors and thus their linkage to injury events would have further benefited their application as per previous work [44]. While the match events included in the current work are self-explanatory in nature, UEFA have yet to publish any detailed operational definitions on the variables of interest and this is an obvious shortcoming that should be acknowledged. This investigation also lacked any insights into short term performance changes across tournament stages and long-term trends from multiple UEFA Euro competitions that may indicate how selected roles are evolving due to modern tactical developments. Finally, as data were individually clipped and then re-filtered from raw sources based on the present studies inclusion criteria and filtering specifications; some differences may be evident compared to UEFA trends [27].

## CONCLUSIONS

The present data demonstrated the match demands placed on players in highly specialised positions during a recent international tournament. Despite substantial performance variation, unique position-specific trends were still found across both volume and intensity metrics. This further supports the use of specialised positional labels when quantifying match demands. These findings could be useful for understanding the specialised demands of football and may provide a basic framework for position-specific drill development. Although practitioners must be aware of contextualising these positional trends based on their players individual qualities and the team’s unique style of play.
